# Supplementary material for: Increased functional connectivity of thalamic subdivisions in patients with Parkinson’s disease
Source: PLoS One. 2019 Sep 4;14(9):e0222002. doi: 10.1371/journal.pone.0222002 (PMC6726201; doi:10.1371/journal.pone.0222002)
Supplement: S1 Text — Full description of the methods used for manual segmentation of the thalamus, independent component analysis denoising, atlas-based region-of-interest segmentation statistics, individual-level general linear model functional connectivity analyses and SPHARM-PDM shape analyses. (DOCX) [file pone.0222002.s001.docx]

**Supporting Information Text**

Increased functional connectivity of thalamic subdivisions in patients with Parkinson’s disease

Conor Owens-Walton, David Jakabek, Brian D. Power, Mark Walterfang, Dennis Velakoulis, Danielle van Westen, Jeffrey C.L. Looi, Marnie Shaw and Oskar Hansson.

**Manual segmentation of the thalamus**

A neuropsychiatrist with extensive experience in the clinical neuroanatomy of the thalamus (BDP) provided training for the CO-W to conduct the manual tracing. Tracing was performed in the coronal plane from the caudal to rostral direction. Neuroradiological and neurosurgical atlases were used to assist with boundary definition and troubleshooting [1, 2]. The method [3] stipulates that the caudal border of the structure encompasses the pulvinar, where the thalamus emerges under the crus fornix. The medial border was defined using various nuclei acting as landmarks, characteristic white matter tracts and in rostral slices the hypothalamic sulcus. The superior border was defined by the body of the lateral ventricle and the rostral border was defined by the diminutive thalamic mass in comparison with the hypothalamus, mamillary bodies, column of the fornix and the optic tract. The protocol excludes the ventral thalamus, the components of which provide helpful landmarks for lateral and inferior boundary delineation. The lateral geniculate nuclei were also excluded from tracing to allow for effective morphological surface mapping to be performed. Reliability of the manual tracing was quantified by the calculation of an *inter*-rater reliability statistic (intraclass correlation coefficient, two-way mixed effects model for absolute agreement), measuring the tracers agreement with BDP (left thalamus, *r* = 0.908 & right thalamus *r* = 0.888), and also an *intra*-rater reliability coefficient statistic (as an intraclass correlation coefficient, two way mixed effects model for absolute agreement), measuring the consistency of the rater over time (left thalamus, *r* = 0.940 & right thalamus *r* = 0.879) [4]. The *inter*-rater score was the final correlation coefficient obtained by CO-W after a significant period of training on an MRI dataset under the training and guidance of BDP. The *intra*-rater score was obtained by CO-W by re-tracing every ~10^th^ participant’s thalamus (bilaterally) during the manual segmentation process, with obtained volumes from the first run correlated against the re-traced volumes.

**Independent component analysis denoising**

A probabilistic independent component analysis (ICA) [5] was performed using the MELODIC tool (Multivariate Exploratory Linear Decomposition into Independent Components, version 3.15). This involves masking of non-brain voxels, voxel-wise de-meaning of the data and normalisation of the voxel-wise variance. Data were whitened and projected into a 53-dimensional subspace using probabilistic principal component analysis where the number of dimensions was estimated using the Laplace approximation to the Bayesian evidence of the model order [5, 6]. The whitened observations were decomposed into sets of vectors which describe signal variation across the temporal domain (time-courses) and across the spatial domain (maps) by optimising for non-Gaussian spatial source distributions using a fixed-point iteration technique [7]. Estimated component maps were divided by the standard deviation of the residual noise and thresholded by fitting a mixture model to the histogram of intensity values [5]. This ICA analysis allows for the identification and removal of motion, scanner and cerebro-spinal fluid artefacts (collectively referred to as noise) using ICA ‘data denoising’ conducted with FSL’s FIX classifier [8]. The FIX classifier used the ‘*Standard.RData trained-weights*’ classifier file which is designed for use on standard functional datasets. The classified ran over a sample of subjects comprised of participants form both PD and Control groups (*n* = 20). The classification of ‘signal’ and ‘noise’ components was checked using the validated guidelines with a conservative approach was taken to any reclassification of ICAs, whereby components identified by the FIX classifier are left in the data unless they are clearly artefactual [9]. Once this checking and reclassification was finished, the FIX classifier was re-trained using this new information and was then run over all participant ICA datasets, regressing out noise components while also cleaning up motion confounds (24 regressors). From this process FIX creates a de-noised filtered functional file that is used for individual and group-level statistical analysis.

**Atlas-based ROI segmentation statistics**

Using FSLStats we calculated the number of voxels that comprised the thalamic seed-ROIs in PD (VLp/VA, M = 185.75; S.D = 17.15; MD/A, M = 546.53; S.D = 46.52) and Controls (VLp/VA , M = 185.50; S.D = 17.67; MD/A, M = 539.75; S.D = 43.52). We then compared these metrics between the two groups using an independent samples t-test and found no significant difference between-groups in voxel numbers for the VLp/VA (*p =* 0.960) or MD/A (*p* = 0.603) seed-ROIs.

**Individual-level GLM functional connectivity analyses**

Three masks were created to cover white matter, ventricle and whole-brain regions, with timeseries extracted from these regions to be regressed from the data at the individual-subject level. These masks were produced using segmentations derived from subjects T1-weighted structural images using FreeSurfer recon-all command [10]. To register these masks for use with FSL, the highres T1 image from each FreeSurfer analysis was registered to each participant’s T1-weighted structural image used in FSL. Average timeseries from within these masks were then calculated using fslmeants. To perform whole-brain signal regression we calculated the average signal across all voxels within a whole brain mask (white matter and grey matter) created using masks from Freesurfer recon-all. These masks were registered to highres space, then to lowres functional space where they were thresholded to only include voxels with a greater than 70% chance of inclusion. This was decided upon based on trial and error, with this number fitting the brain tissue most comprehensively whilst still excluding voxels from ventricle and CSF regions. We then used fslmeants to extract the average signal within this mask to be regressed from the data. All masks were also eroded using fslmaths to avoid partial volume effects.

We incorporated a temporal derivative into the design matrix for each individual-level GLM to shift the model in time and account for variations in the hemodynamic response function and slice timing delays, allowing for a better fit for the whole model, reducing unexplained noise and potentially increasing statistical significance. The temporal derivative is based upon 1^st^ order Taylor series expansion where f$\left( t+a \right)\approx f\left( t \right)+a.f'(t)$. In this function, f$\left( t+a \right)$ represents the shifted EV, $f\left( t \right)$ represents the EV and $a.f'(t)$ represents the temporal derivative. We also removed timepoints corrupted by large motion-related signal by calculating the temporal derivative of timecourses for root mean squared head position change for volume N to volume N+1 (DVARS) [11]. This was performed using FSL Motion Outliers which identifies timepoints with significant DVARS outliers (75th percentile plus 1.5 multiplied by the interquartile range). This approach can help deal with large motion effects that are unable to be resolved by traditional linear motion parameter regression methods [12]. Neuroanatomical labelling of significant results used the Harvard-Oxford Cortical and Subcortical Atlases within FSL, incorporating the Yale BioImage Suite MNI to Talairach and Brodmann Areas online converter tool (<https://medicine.yale.edu/bioimaging/suite/>).

**SPHARM-PDM detail**

Using the 3-D binary object maps obtained from the manual region-of-interest segmentation of the thalamus, shape analysis was performed using the spherical harmonic toolkit SPHARM-PDM [13]. Segmentations were pre-processed to ensure a spherical topology, then described by spherical harmonic functions and sampled onto surfaces of 1002 points. Surfaces were then aligned using a rigid-body Procrustes alignment to a mean template created from a group sample. Comparisons between groups were performed using multivariate analysis of covariance models with a Hotelling statistic in R (version 3.2.1, R Development Core Team, 2014). Significant surface change was displayed at *p* < 0.05 with a correction for multiple comparisons performed using a false-discovery rate estimation of *q* = 5% [14]. All analyses included covariates of intracranial volume, age and sex. Statistical shape analysis provides visualisations of the local effect size via mean difference magnitude displacement maps. Mean difference displacement maps display the magnitude of surface change (deflation or inflation) in mm between corresponding points on the mean surfaces of PD subjects relative to the Control group.

**References**

1. Duvernoy HM. The human brain: surface, three-dimensional sectional anatomy with MRI, and blood supply: Springer Science & Business Media; 2012.

2. Morel A. Stereotactic atlas of the human thalamus and basal ganglia: CRC Press; 2007.

3. Power BD, Wilkes FA, Hunter-Dickson M, van Westen D, Santillo AF, Walterfang M, et al. Validation of a protocol for manual segmentation of the thalamus on magnetic resonance imaging scans. Psychiatry Research: Neuroimaging. 2015;232(1):98-105.

4. Shrout PE, Fleiss JL. Intraclass correlations: uses in assessing rater reliability. Psychological bulletin. 1979;86(2):420.

5. Beckmann CF, Smith SM. Probabilistic independent component analysis for functional magnetic resonance imaging. IEEE transactions on medical imaging. 2004;23(2):137-52.

6. Minka TP, editor Automatic choice of dimensionality for PCA. Advances in neural information processing systems; 2001.

7. Hyvarinen A. Fast and robust fixed-point algorithms for independent component analysis. IEEE transactions on Neural Networks. 1999;10(3):626-34.

8. Salimi-Khorshidi G, Douaud G, Beckmann CF, Glasser MF, Griffanti L, Smith SM. Automatic denoising of functional MRI data: combining independent component analysis and hierarchical fusion of classifiers. Neuroimage. 2014;90:449-68.

9. Griffanti L, Salimi-Khorshidi G, Beckmann CF, Auerbach EJ, Douaud G, Sexton CE, et al. ICA-based artefact removal and accelerated fMRI acquisition for improved resting state network imaging. Neuroimage. 2014;95:232-47.

10. Fischl B. FreeSurfer. Neuroimage. 2012;62(2):774-81.

11. Smyser CD, Inder TE, Shimony JS, Hill JE, Degnan AJ, Snyder AZ, et al. Longitudinal analysis of neural network development in preterm infants. Cerebral cortex (New York, NY : 1991). 2010;20(12):2852-62.

12. Power JD, Barnes KA, Snyder AZ, Schlaggar BL, Petersen SE. Spurious but systematic correlations in functional connectivity MRI networks arise from subject motion. Neuroimage. 2012;59(3):2142-54.

13. Styner M, Oguz I, Xu S, Brechbuhler C, Pantazis D, Levitt JJ, et al. Framework for the Statistical Shape Analysis of Brain Structures using SPHARM-PDM. The insight journal. 2006(1071):242-50.

14. Genovese CR, Lazar NA, Nichols T. Thresholding of statistical maps in functional neuroimaging using the false discovery rate. Neuroimage. 2002;15(4):870-8.
