# Supplementary material for: Increased functional connectivity of thalamic subdivisions in patients with Parkinson’s disease
Source: PLoS One. 2019 Sep 4;14(9):e0222002. doi: 10.1371/journal.pone.0222002 (PMC6726201; doi:10.1371/journal.pone.0222002)
Supplement: S1 Table — All values are rounded to two decimal places. VLp/VA thalamus, clusters 1–6, MD/A thalamus clusters 7–9. Significance based on a two-tailed Pearson correlation controlling for age, sex and years of education. A correction for multiple comparisons using the Bonferroni method stipulate a p-value of < 0.000925 required for significance (based on performing 54 analyses). LED, levodopa equivalent dosage; UPDRS-III, Unified Parkinson’s Disease Rating Scale part III; TUG, Timed Up and Go test; AQT, A quick test of cognitive speed test; AF, Animal fluency test. (DOCX) [file pone.0222002.s002.docx]

**S2 Table. Correlations between functional connectivity and clinical data.**

| **Maxima number** | **LED** | | **Disease duration** | | **UPDRS-III** | | **TUG** | | **AQT** | | **AF** | |
| --- | --- | --- | --- | --- | --- | --- | --- | --- | --- | --- | --- | --- |
|  | **r** | ***p*** | **r** | ***p*** | **r** | ***p*** | **r** | ***p*** | **r** | ***p*** | **r** | ***p*** |
| **1** | 0.32 | 0.13 | 0.41 | 0.03 | 0.12 | 0.52 | 0.23 | 0.37 | 0.18 | 0.17 | 0.26 | 0.23 |
| **2** | 0.31 | 0.13 | 0.32 | 0.09 | 0.11 | 0.57 | 0.43 | 0.02 | 0.20 | 0.31 | 0.25 | 0.18 |
| **3** | 0.49 | 0.01 | 0.57 | 0.01 | -0.04 | 0.82 | 0.13 | 0.52 | 0.18 | 0.37 | 0.18 | 0.35 |
| **4** | -0.41 | 0.04 | -0.21 | 0.28 | -0.03 | 0.88 | -0.13 | 0.52 | -0.13 | 0.52 | -0.02 | 0.93 |
| **5** | -0.33 | 0.11 | -0.18 | 0.36 | 0.33 | 0.08 | 0.05 | 0.82 | -0.04 | 0.83 | 0.11 | 0.58 |
| **6** | -0.36 | 0.08 | -0.31 | 0.11 | 0.10 | 0.60 | 0.43 | 0.02 | -0.08 | 0.69 | 0.08 | 0.68 |
| **7** | -0.20 | 0.34 | -0.28 | 0.14 | 0.04 | 0.86 | 0.29 | 0.13 | 0.16 | 0.42 | 0.08 | 0.68 |
| **8** | 0.16 | 0.46 | 0.30 | 0.12 | 0.19 | 0.32 | 0.27 | 0.16 | 0.14 | 0.48 | 0.33 | 0.08 |
| **9** | -0.28 | 0.17 | -0.19 | 0.31 | 0.08 | 0.68 | 0.37 | 0.05 | -0.01 | 0.97 | 0.03 | 0.90 |
| All values are rounded to two decimal places. VLp/VA thalamus, clusters 1-6, MD/A thalamus clusters 7-9. Significance based on a two-tailed Pearson correlation controlling for age, sex and years of education. A correction for multiple comparisons using the Bonferroni method stipulate a *p*-value of < 0.000925 required for significance (based on performing 54 analyses). LED, levodopa equivalent dosage; UPDRS-III, Unified Parkinson’s Disease Rating Scale part III; TUG, Timed Up and Go test; AQT, A quick test of cognitive speed test; AF, Animal fluency test. | | | | | | | | | | | | |
