# Supplementary material for: Increased functional connectivity of thalamic subdivisions in patients with Parkinson’s disease
Source: PLoS One. 2019 Sep 4;14(9):e0222002. doi: 10.1371/journal.pone.0222002 (PMC6726201; doi:10.1371/journal.pone.0222002)
Supplement: S2 Table — A correction for multiple comparisons using the Bonferroni method stipulate a p-value of < 0.00625 required for significance (based on performing 8 analyses). UPDRS-III, Unified Parkinson’s Disease Rating Scale part III; TUG, Timed up and Go test; AQT, A Quick Test of Cognitive Speed; AF, Animal Fluency test; R2 change, variance in clinical measure score explained by unique contribution of the volume of interest (multiply by 100 to find percentage); β, standardized beta coefficient, indicating effect size. (DOCX) [file pone.0222002.s003.docx]

**S3 Table.** **Correlations between thalami volumes and clinical measures: PD and Controls.**

| **Structure** | **Group** | **Clinical measure** | **R^2^**  **change** | **β** | ***p*-value** |
| --- | --- | --- | --- | --- | --- |
| Right thalamus | Controls | UPDRS-III | 0.001 | 0.041 | 0.906 |
|  | PD |  | 0.006 | 0.149 | 0.671 |
| Left thalamus | Controls |  | 0.07 | 0.371 | 0.266 |
|  | PD |  | 0.002 | 0.062 | 0.802 |
| Right thalamus | Controls | TUG | 0.067 | -0.366 | 0.284 |
|  | PD |  | 0.062 | 0.463 | 0.128 |
| Left thalamus | Controls |  | 0.007 | -0.118 | 0.732 |
|  | PD |  | 0.021 | 0.192 | 0.380 |
| Right thalamus | Controls | AQT | 0.001 | 0.037 | 0.909 |
|  | PD |  | 0.016 | 0.232 | 0.515 |
| Left thalamus | Controls |  | 0.049 | -0.326 | 0.315 |
|  | PD |  | 0.038 | 0.257 | 0.306 |
| Right thalamus | Controls | AF | 0.011 | -0.152 | 0.655 |
|  | PD |  | 0.098 | 0.584 | 0.135 |
| Left thalamus | Controls |  | 0.047 | -0.318 | 0.358 |
|  | PD |  | 0.077 | 0.367 | 0.188 |
| A correction for multiple comparisons using the Bonferroni method stipulate a *p*-value of < 0.00625 required for significance (based on performing 8 analyses). UPDRS-III, Unified Parkinson’s Disease Rating Scale part III; TUG, Timed up and Go test; AQT, A Quick Test of Cognitive Speed; AF, Animal Fluency test; R^2^ change, variance in clinical measure score explained by unique contribution of the volume of interest (multiply by 100 to find percentage); β, standardized beta coefficient, indicating effect size. | | | | | |
